# [Retracted] Effects and mechanisms of fatty acid metabolism-mediated glycolysis regulated by betulinic acid-loaded nanoliposomes in colorectal cancer

**DOI:** 10.3892/or.2025.8994

**Published:** 2025-09-23

**Authors:** Gang Wang, Yang Yu, Yu-Zhu Wang, Zhi-Min Zhu, Pei-Hao Yin, Ke Xu

Oncol Rep 44: 2595–2609, 2020; DOI: 10.3892/or.2020.7787

Following the publication of this paper, it was drawn to the Editor's attention by a concerned reader that, regarding the western blot experiments shown in Figs. 6E and 7C, a pair of the gel slices were strikingly similar in appearance, suggesting that the same data had been included in these figures to show the results with different proteins. Moreover, two additional gel slices in these figures had apparently been re-used in a pair of subsequent papers published by the same research group, where the experimental conditions were indicated to be different, and it was noted by the Editorial Office that one of the gel slices may have contained an anomaly in the form of a break in the continuity of the gel.

Upon asking the authors to provide the Editorial Office with an explanation of the above issues, they did supply us with revised versions of Figs. 6E and 7C; however, it was subsequently pointed out to the authors that certain of the western blot data in Fig. 6E had apparently reappeared in a further paper by the same authors, in the journal *Integrative Cancer Therapies*. Owing to the lack of a further response from the authors in relation to the latter query, the Editor of *Oncology Reports* has decided that this paper should now be retracted from the Journal on account of a lack of confidence in the presented data. The Editor apologizes to the readership for any inconvenience caused.

